# Failed Implementation of Mobile Access to Electronic Health Records in Home Care: Qualitative Study in Sweden

**DOI:** 10.2196/69590

**Published:** 2026-01-23

**Authors:** Lovisa Jäderlund Hagstedt, Helena Hvitfeldt, Maria Hägglund

**Affiliations:** 1 Department of Learning, Informatics, Management and Ethics, Karolinska Institutet, Stockholm, Sweden; 2 Tiohundra AB, Stockholm, Sweden; 3 Department of Women's and Children's Health, Uppsala University, Box 256, Uppsala, 751 85, Sweden, +46(0)729999381; 4 Uppsala University Hospital, Uppsala, Sweden

**Keywords:** home care, primary care, work environment, CFIR, mHealth, eHealth, qualitative evaluation, electronic health record

## Abstract

**Background:**

Digitalization and mobile health (mHealth) technologies hold promise for improving home care delivery. However, many mHealth initiatives fail to achieve their goals. Understanding the reasons behind these failures is critical for informing the successful implementation of mHealth in primary and home care settings.

**Objective:**

This study aimed to explore the implementation process of a tablet computer with an mHealth app providing mobile access to the electronic health record (EHR) in home care, identifying barriers and facilitators to its uptake.

**Methods:**

A tablet with EHR access was introduced at 4 primary care centers and 1 municipal home care organization in Sweden. Participants were nurses and physicians working at the study sites. Focus group discussions and interviews were conducted to obtain a rich understanding of implementation-related issues experienced by the health care professionals. Qualitative content analysis was conducted using the Consolidated Framework for Implementation Research to guide interpretation.

**Results:**

Eighteen health care professionals (16 nurses and 2 physicians) participated in the study. The implementation of the mHealth app was largely unsuccessful. Key barriers included limited functionality of the app, technological immaturity, and unstable infrastructure. Organizational context influenced uptake, especially due to differing EHR systems and varying levels of user engagement. Users who were involved in the development process were more positive, despite the absence of certain functionalities, while those excluded struggled with adoption. Long development and implementation timelines and limited training reduced enthusiasm and negatively affected user engagement. Additional challenges included insufficient implementation planning, lack of leadership engagement, and inadequate resources for support and training.

**Conclusions:**

For mHealth implementations to succeed, tools must meet users’ needs and integrate seamlessly with existing eHealth ecosystems and infrastructures. Premature implementations can lead to change fatigue and diminish future engagement. Investments in user-centered design, thorough testing, organizational readiness, and sustained support are essential to realize the potential of mHealth in home care.

## Introduction

As populations age, the need for primary care and home care increases [1], yet resources remain scarce, with serious recruitment challenges concerning educated personnel [2,3]. According to the Swedish Association of Local Authorities and Regions, in the future, such care must be provided closer to the patients. Further, a holistic approach, greater use of digitalization, more collaboration among departments and specialties, and improved patient access are considered necessary. This sentiment is echoed in the World Health Organization’s global strategy on digital health, where the purpose of the strategy is to “strengthen health systems through the application of digital health technologies for consumers, health professionals, health care providers and industry towards empowering patients and achieving the vision of health for all” [4]. Electronic health records (EHRs) are used in everyday care throughout Sweden [5]. Most regions use one EHR system throughout hospitals, primary care, and psychiatric care, making patient records available in the entire region. Elder care, however, is the responsibility of municipalities that fall under different legislation [6], and often use separate EHR systems [7-9].

The concept of mobile health (mHealth) has emerged as a subcategory of eHealth and can be defined as medical and public health practice supported by mobile devices [5,10]. The use of smartphones and tablets in health care is increasing. The use of mobile devices for home care in Swedish municipalities has increased significantly in the past few years; in 2020, 41% of the municipalities provided health care staff the possibility to read and write information using a mobile device, compared to 5% in 2015 [11,12]. By 2024, 70% of the municipalities used mobile devices in home care [12].

Further digitalization and the introduction of eHealth and mHealth have been seen as means to optimize health care [4]. Expectations are high that they will be the solution to many of the problems that exist in health care today [13]. However, many projects are unsuccessful [14], and challenges of digitalization have been raised [15]. There are several reasons why implementations of eHealth in health care fail [16,17]. By studying and analyzing why an eHealth or mHealth implementation is unsuccessful, we could learn how successful digitalization in primary and home care can be achieved.

A Cochrane review of mHealth apps showed that the complexities of health care delivery and human interactions defy simplistic conclusions on how health workers will perceive and experience their use of mHealth. Perceptions reflect the interplay between technology, contexts, and human attributes. Detailed descriptions of the program, implementation processes, and contexts, alongside effectiveness studies, will help to unravel this interplay to formulate hypotheses regarding the effectiveness of mHealth [18]. Despite the increasing use of mHealth [19], limited research on mobile EHRs exists, mostly focusing on in-hospital use for bedside EHR access [20,21]. Studies indicate that usability issues of mobile EHRs combined with low digital literacy negatively impact nurses’ adoption of the tools [21], and may even have a negative impact on nurses’ work environment despite the aim to better support their work [22].

This study was designed to evaluate an mHealth app that provides health care professionals (HCPs) with mobile access to the EHR and describe its impact on work processes and work environment. The project was, however, terminated prematurely since HCP did not adopt and use the system. The collected data were instead analyzed to understand the reasons for the implementation failure.

We aim to understand the reasons for an unsuccessful mHealth implementation and potential success factors by describing lessons learned from the implementation process of a tablet computer with an mHealth app that provides mobile access to EHR in home care.

## Methods

### Overview

A qualitative approach has been applied to understand the HCPs’ expectations before implementation and their experiences of using the system. Individual interviews and focus group interviews have been used to obtain a rich understanding of the implementation-related issues. Due to the limited number of users of the mHealth app, a qualitative approach was deemed suitable to gain an in-depth understanding of the factors causing the failed implementation. The Consolidated Framework for Implementation Research (CFIR) by Damschroder et al [23] was used to guide our analysis.

### Study Setting

The study was conducted in a public municipality home care organization (MHCO) and the home care sections of 4 public academic primary care centers (PCCs) in a rural part of Region Stockholm.

The home care organization provides medical and supportive care for a diverse range of patients, predominantly older adults but also younger individuals with medical needs. The MHCO has the main responsibility for home care, whereas the PCCs provide some home care, for example, the first 2 weeks after discharge from the hospital and the support and service for persons covered under the Swedish Act Concerning Support and Service for Persons with Certain Functional Impairments. At the time of this study, there was no interaction between the PCCs and the MHCO.

**Figure 1.**
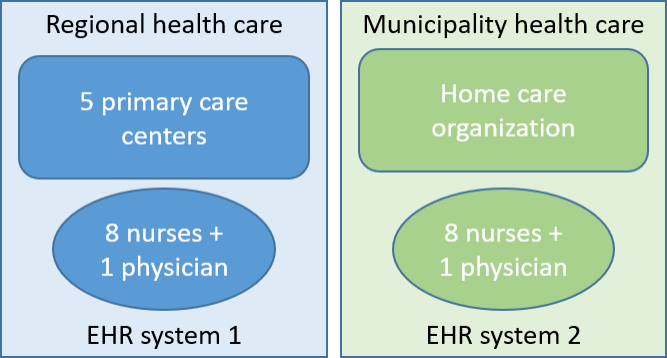
Study setting describing the regional and municipality settings and included study participants. EHR: electronic health record.

The PCCs all use the same EHR system, accessible through the mHealth app, whereas the MHCO has a different EHR system, lacking accessibility through the mHealth app. Figure 1 presents an overview of the study setting.

### The Intervention

The intervention consists of a tablet computer with several apps (eg, decision support, online prescription tools, and online supplies ordering), and the new mHealth app that provides mobile access to the EHR (example screenshots from the app are in Multimedia Appendix 1). In the following text, we will refer to the latter as “the mHealth app” and use the term “the mobile tool” when referring to the tablet with all its apps.

The mHealth app on the tablet computer was developed after an initiative by professionals (2 doctors and 2 nurses) at one of the participating academic PCCs. They saw a need for easy access to information, including patient data, and to carry out all parts of their home care work, including documentation, while in the patient’s home. The HCP took an active part in an initial need and requirement analysis, and the mHealth app was then developed from their ideas. The first author was involved in some of the app’s needs and requirement analysis. Professionals provided feedback on functionality and design but were not further involved in the mHealth app’s development. No structured usability assessments were performed.

The project was carried out through a collaboration between academic PCCs and the IT unit of Region Stockholm, along with the private company Chorus, which was responsible for the technical development of the app. As an innovation project, each participating entity covered its own costs. The aim of the pilot project was to evaluate the tool’s effectiveness and its potential for broader implementation within the region, with the long-term goal of generating both clinical benefits and economic value. The mobile tool was tested at selected academic PCCs, and the responsibility for approval and rollout was shared between the involved academic PCCs and the IT unit of Region Stockholm.

The mHealth app provided mobile access to the EHR used in regional home care. This included, for example, access to patient record entries, information about the patients to be visited during the day, and the possibility to write patient record entries while in the patient’s home, a task otherwise done after returning to the office, as previously described in our study outlining the matching of the functionality of the app to the home care work processes [24]. While the previously published study described the work process in primary care in detail, matching it to the functionality of the study, it did not go into details on the users’ experiences and reasons for the failed implementation. Due to technical difficulties, not all parts of the EHR were accessible through the mHealth app. The app did not provide information on patients’ medications, referrals, or radiology results because the EHR from which the mHealth app collected the medical data did not support this output. However, it was possible to access information on the patients’ previous outpatient and inpatient care visits, diagnoses, and laboratory tests. Thereby, some, but not all the HCPs’ needs were met by the mHealth app. The mHealth app was intended to be further developed, and the implementation was therefore initiated.

The tablet computer also provided access to several clinical decision support systems and a mobile web-based prescription system that is used for some of the patients in home care, thus enabling the HCP to manage part of those patients’ medications. Thereby, the diverse functions of the tablet computer provided HCPs with a multifaceted mobile tool for home care visits.

There were no costs involved for the users. Use of the mobile tool was strongly encouraged but voluntary.

The mobile tool was first implemented in the home care section of the PCC, which was part of the development of the mHealth app and in one of the other PCCs. In both participating PCCs where the mobile tool was first implemented, some HCPs were avid advocates for the mHealth app.

At a later stage, the mobile tool was implemented in the MHCO and the remaining PCCs. There were no advocates for the mHealth app at either of these workplaces. The mHealth app was further developed later to improve stability and implemented and used in in-hospital care for bedside record access in the Stockholm region. In 2020, Region Stockholm ceased all support of the mHealth app, and it is currently not in commercial operation.

### Data Collection

Data for this study were collected through focus groups and semistructured interviews with nurses and doctors working in home care where the mobile tool was introduced. The first author (LJH, female) was responsible for the data collection, with the assistance of 2 Master’s students (both female, health informatics students). All 3 had both training in and experience of qualitative data collection, and were supported by the last author (MH, female) with extensive experience in qualitative research. LJH was working as a general practitioner in the region at the time of the study and was well-known to all study participants. An overview of the timeline and data collection is presented in Table 1. Before implementation, a workshop was held where questions were asked regarding the time spent on administrative work related to home visits, experienced stress, use of decision support tools, and the experienced quality of documentation and communication between HCPs.

**Table 1.** Overview of the data collection timeline, including the method used, setting, and time for data collection.

Data collection	PCC1^a^	PCC2	PCC3	PCC4	MHCO^b^
Development of an app	2014‐2015	—^c^	—	—	—
Focus group on expectations	November 2015	May 2016	April 2017	April 2017	April-May 2017
Individual interviews	May 2016	February 2017	February 2018	March 2018	February 2018
Focus group on completion of study	October 2016	—	—	—	—

a PCC: primary care center.

b MHCO: municipality home care organization.

c No data were collected using that method at that specific unit.

The mobile tool was introduced and demonstrated during a workshop where participants had the opportunity to practice using it. The demonstration was conducted by guiding participants through the different functionalities step by step, ensuring a comprehensive understanding of its features and applications. This hands-on approach facilitated engagement and allowed attendees to gain practical experience with the tool.

The original plan was to conduct data collection through (1) preimplementation focus groups, (2) individual interviews after 6 months of use, and (3) a second round of focus group interviews after 12 months of use. The different qualitative methods were chosen to gather a rich dataset; the focus group interviews enabled discussion and exchange of opinions between participants, whereas the interviews allowed more detailed exploration of individual HCPs’ experiences of using the mobile tool. We also recognized that individual interviews provide an opportunity for participants to express thoughts and concerns they might be reluctant to share in a focus group setting with other participants present.

Interview guides were developed for individual and focus group interviews (MultimediaAppendices 2  3). All interviews and focus groups were recorded and transcribed. Notes were also taken during focus groups and included in the material. All interviews were held in Swedish. The individual interviews lasted 20‐30 minutes and the focus group interviews lasted 1‐1.5 hours. Data collection was conducted where most convenient for the participants, that is, at the PCCs or the MHCO’s office. Data collection was conducted during working hours. Participant checking of the transcribed interviews was not done to reduce the burden on the study participants.

Preimplementation focus group interviews were held in all the PCCs and the MHCO, where the positive and negative expectations were discussed and reflected upon by HCP who would use the mobile tool.

Individual interviews with nurses and physicians were conducted according to plan to capture the staff’s experiences using the mobile tool and its impact on work processes.

Interviews were conducted when the mobile tool had been used for at least 6 months. All interviews were conducted by the first author and the Master’s students in the research team. The Master’s students conducted interviews in the MHCO. One of the Master’s students continued working in the project as a research assistant after graduation and also contributed to the data analysis.

All eligible nurses and physicians working in the setting were invited and accepted to participate in the study. Study participants were approached via email and in-person by the first author through the managers of the MHCO and PCCs. The same participants at each site participated in both the focus groups and interviews.

A second set of focus group interviews was planned at the end of the study, when the mobile tool had been used for 1 year. The PCC involved in the mHealth app’s development started testing earlier than the other clinics, and a focus group interview was held a year after they started using the mobile tool to capture their long-term experiences. When the 6-month interviews were conducted at the other clinics, they had stopped using the mobile tool, and the decision was made not to conduct the planned focus group interviews, as they would not gain further experiences of using the mobile tool.

### Data Analysis

All interviews were transcribed verbatim and analyzed using a deductive content analysis approach [25]. The CFIR [23] was used to structure the analysis. The data analysis performed in this study does not include any quantification of responses regarding specific categories.

CFIR identifies 5 domains that are essential to implement an intervention [23]:

1. Intervention characteristics—features of the intervention itself, such as complexity, evidence strength and quality, and adaptability.
2. Outer setting—the external environment influencing implementation, such as external policies and incentives.
3. Inner setting—organizational factors such as culture, leadership, and available resources within the implementing organization.
4. Characteristics of individuals—traits and actions of the individuals involved in the implementation, including knowledge, beliefs, and self-efficacy.
5. Process—the planning, execution, and reflection processes involved in implementing the intervention.

**Figure 2.**
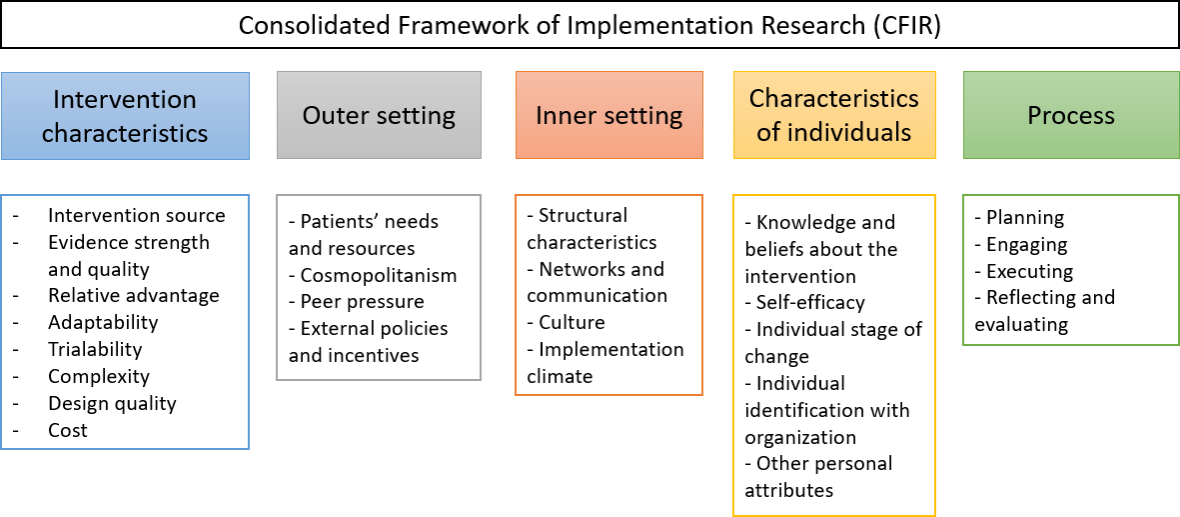
An overview of the Consolidated Framework for Implementation Research, including the 5 domains and their constructs.

In CFIR, a domain represents a broad category of factors or determinants that affect the implementation process. A construct, on the other hand, refers to a specific factor within a domain that provides more detailed insight into the aspects of implementation. An overview of the CFIR domains and their constructs is presented in Figure 2.

The 3 authors all actively participated in the analysis. Initial coding was performed by LJH, MH, and one of the Master’s students who continued working on the project as a research assistant after graduating. First, all materials were read and reread, and meaning-bearing units were identified. Each coder coded and categorized the meaning-bearing units individually into 1 of the 5 domains of the CFIR framework. HH joined the analysis at this stage, and the 3 authors discussed the coding until a consensus was reached on the interpretation. Where appropriate, relevant CFIR constructs were used to signify our findings, but we did not systematically search for constructs in the material.

Due to the relatively small number of participants, achieving full data saturation was not possible. However, we ensured sufficient information power by including all eligible HCPs across multiple roles and settings. Thematic consistency across discussions suggests key insights were captured despite the limited number of study participants.

### Ethical Considerations

The Research Ethics Committee at Karolinska Institutet has granted ethical approval for this study (DNR 2015/1457-31/5). All study participants provided written or oral informed consent before participating in the study. In accordance with the Swedish Ethical Review Authorities’ requirements, participants were informed that their participation was voluntary and that they could choose to revoke their participation at any time. Transcribed interviews and focus groups were deidentified to protect the participants’ privacy, and confidentiality was preserved when reporting results. Study participants did not receive any compensation for their time.

## Results

### Overview

The 18 participants in the study were all nurses and physicians working in home care in either the home care section of the participating PCCs or the MHCO. A total of 8 nurses and 1 physician from the PCCs (PCC1=2 nurses and 1 physician, PCC2=1 nurse, PCC3=3 nurses, and PCC4=2 nurses), and 8 nurses and 1 physician from the MHCO participated in the study (Figure 1). Participants were predominantly female (17/18, 94.5%), and age ranged between 43 and 64 years. Due to the small number of participants in the study, we chose not to collect and report further detailed characteristics of the individual study participants to ensure their anonymity.

Using the 5 domains of CFIR, we present our analysis of the HCPs’ expectations before implementation and their experiences during the implementation of the mobile tool. We identify the key barriers causing the implementation to fail and the aspects that had a positive influence.

### Intervention Characteristics

Before the implementation, HCPs’ expectations were high, yet the interviews after 3 and 6 months of using the app revealed that many of the expectations were unmet. Textbox 1 presents an overview of the constructs related to the characteristics of the intervention, with further details from the qualitative analysis presented below.

Textbox 1. **Identified categories related to the Consolidated Framework for Implementation Research domain intervention characteristics, divided by expectations before and experiences after implementation.**

**Expectations before implementation**

- Relative advantage
  - Reduction of cognitive workload
  - Time-efficient and timely
  - Real-time access to information
  - Lack of information and functionality
- Complexity
  - Increased complexity and double documentation with many mobile apps
  - Workarounds to adapt to the system may increase cognitive workload
- Design quality
  - Slow and frustrating
  - Immature and unstable

**Experiences after use**

- Relative advantage
  - Reduction of the cognitive workload
    - Finish work on the site
    - No need for reminders
  - Access to important functions and information
  - Missing functions
  - Reduces unnecessary lab tests and orders
  - Note formatting is worse
- Complexity
  - Adaptations of work processes are required
    - Condensed documentation
    - Preliminary documentation on site
- Design quality
  -  Poor design or unclear layout
  -  Design improved during the project
  -  Immature and unstable

#### Preimplementation Expectations

HCP had high hopes that the mobile tool would support their work and serious concerns that the technology would not meet their expectations.

##### Relative Advantage

During the preimplementation focus group interviews, the HCP expressed high expectations that the new mobile tool would help reduce their cognitive workload by allowing them to complete tasks (eg, ordering materials or sending referrals) and finish documentation at the patients’ home:

*To complete the documentation before leaving the patient’s home. It would be a dream. You forget so much on the way back to the office.*

[Nurse, MHCO]

Real-time access to information would make it possible to make more informed decisions and reduce the need to call colleagues to find information, for example, from the EHR. However, there were also concerns:

*It’s faster to write a post-it note. If it [the mobile tool] doesn’t provide everything we need, it may end up in the office*

[Nurse, PCC 1]

Staff already knew that some important functionality (eg, the medication list and referrals) would not be available, and concerns were that this would limit the app’s usefulness:

*Frankly, it feels a bit underdeveloped. Several things are missing, like the medication list and x-ray results, which is important when you visit the patient and need to discuss prior events or plans.*

[Physician, PCC 1]

##### Complexity

There were also concerns that missing functionality would result in workarounds and the need for double documentation that might increase the administrative and cognitive workload, especially concerning medications and the lack of necessary templates in the medical record:

*Above all, I would like access to the medication list. I know I won’t, but it would make my job much easier since I organize the patients’ pill box dispensers in their homes.*

[Nurse, PCC2]

##### Design Quality

Before the implementation, there were some apprehensions regarding the system’s maturity and that it might be slow and frustrating:

...*that the software is only half-developed and not fully functional. (Physician, PCC 1*). Several HCP were worried that the testing would be hampered due to immature technology: *If it’s malfunctional you won’t use it much.*

[Nurse, PCC 1]

### Intervention Experiences

#### Overview

The semistructured interviews and the focus group showed that the users’ needs and requirements were not sufficiently met by the mobile tool, largely because the mHealth app was not sufficiently adapted to the users’ needs. There was, however, a notable difference between how the mobile tool was perceived by the HCP who worked where the intervention was first implemented and who had taken a more active role in the needs analysis and requirements specification, compared to those working where the intervention was later implemented. Those involved in the mHealth app’s development and who were part of the initial implementation considered the app much more useful than those who had not been part of the development.

#### Relative Advantage

One of the nurses responded as follows:

*Patient visits have become more complete, new questions that arise can be answered quickly.*

[Nurse, PCC 1]

The HCP experienced that the mobile tool facilitated work processes and found it fulfilling to be able to answer questions from patients immediately. Another positive effect of the intervention was that it reduced the cognitive workload:

*I don’t need to bring notes that I will later have to remember to document separately on every patient.*

[Nurse, PCC 2]

However, not all needs and requirements were met. The concerns about missing functions that were expressed before the implementation were confirmed. Several of the participants stated that the mHealth app needed further development and additional functions:

*Honestly, it feels undeveloped. Several things are missing, like the medication list and x-ray results, which are important when you’re in the patient’s home.*

[Physician, PPC 1]

#### Complexity

The limitations in functionality, with missing templates, lack of dictation functionality, and technical challenges with the smaller keyboard on the tablet computer, proved to be a confining factor:

*The templates are a bit too, well...wrong. So, it becomes very limited, and then it feels easier to take notes by hand.*

[Nurse, PCC 1]

Many of the HCPs also felt that their documentation became more concise, and that the on-site documentation in the app was preliminary and needed further development when they returned to the office:

*The documentation may be a little shorter, sometimes the record entries become shorter.*

[Nurse, PCC 1]

For some, the limitations in documentation functionality resulted in them not using that at all:

*And still, for me, it is faster to dictate than to write. So, I do not write any record entries in the app. I use it to read record entries in the patient’s home. Then it works well for the most part.”*

[Physician, PCC 1]

#### Design Quality

The users were not impressed by the app’s layout, especially the design of the laboratory results:

*But you get for example all the hemoglobin results one after the other. It’s difficult to read. Seven or eight rows of results for each blood test.*

[Nurse, PCC 1]

The users did, however, feel that the design improved somewhat during the project.

Most of the HCPs experienced difficulties with the functionality and deemed the app immature and unstable.

*Sometimes the internet connection has been bad. Sometimes the application does not work despite an adequate internet connection. It has happened that I have been able to access the medical record, but then it just stops working. I tap and tap but nothing happens. That’s it.*

[Physician, PCC 1]

Another user stated:

*All too often it has not worked. Either the internet connection has been inadequate or there is no explanation why I can’t log in.*

[Nurse, PCC 1]

### Outer Setting

In this domain, the expectations of the intervention coincided with the actual experiences of the intervention to a large extent. Textbox 2 shows the categories identified in the outer setting domain.

Textbox 2. **Identified categories related to the Consolidated Framework for Implementation Research domain outer setting, divided by expectations before and experiences after implementation.**

**Expectations before implementation**

- Technical infrastructure
  - Previous experiences among the staff with poor internet connectivity in the home care areas caused worry
- Patient needs and resources
  - Health care professionals looked forward to being able to use the mobile device as a tool for learning and patient communication
  - Being able to answer the patients’ questions immediately

**Experiences after use**

- Technical infrastructure
  - Poor internet connectivity
- Patient needs and resources
  - Supports better work processes and improves patient safety.
  - Facilitates patient participation.

#### Preimplementation Expectations

HCPs were expressing concerns regarding the internet connectivity in the area but were also looking forward to using the mobile tool to meet the needs of their patients.

##### Technical Infrastructure

Network connectivity is limited in parts of the rural areas of the study setting, and since many of the HCPs had previous experiences with problems due to poor internet connectivity, concerns were expressed before the implementation:

*The fear is the network connection. That’s the thing, and it’s hard to solve.*

[Nurse, PCC 2]

##### Patient Needs and Resources

HCPs were hoping that the mobile tool would enable them to improve their communication with patients as well as the patients’ participation in their care by using it as both a tool for learning:

*And you may be able to show patients how to easily access sites for healthcare e-services*

[Nurse, PCC 1]

and a means to answer questions asked by the patient:

*Medical record entries may be useful when you’re in the patient’s home. Like if a patient asks, ‘ What did the doctor say at the last visit? I did not understand.’*

[Nurse, MHCO]

### Intervention Experiences

The HCP’s expectations regarding the outer setting were fulfilled to a high degree.

#### Technical Infrastructure

The limited network connectivity was a real challenge when using the mobile tool. One of the users stated:

*Our damn bad network connection has proven to be a huge, huge problem.*

[Nurse, PCC 2]

#### Patient Needs and Resources

The staff experienced that the mobile tool supported better work processes and thus improved patient satisfaction:

*communication with the patient runs smoother because I’m able to answer questions on-site, eg, concerning lab results.*

[Nurse, PCC 1]

However, when the mHealth app did not work, it also sometimes rendered other, less positive questions from patients:

*I’ve appreciated being able to answer a question that the patient has asked. But sometimes you get questions about the application when it doesn’t work. A patient asked me - But if it does not work, why do you use it?*

[Physician, PCC 1]

### Inner Setting

#### Overview

All HCPs experienced that the mHealth app was not adapted to their workflow. Therefore, the mobile tool was not seen as entirely useful. This was especially apparent in the MHCO and for the participating physicians since important functions for their workflow were lacking to a larger extent than for the nurses in the PCCs. There were also implications that the implementation process would have benefited from a stronger commitment from the organization and some of the HCPs from the MHCO experienced that there was not enough time for training and preparation before the implementation. Textbox 3 gives an overview of the categories in this domain.

Textbox 3. **Identified categories related to the Consolidated Framework for Implementation Research domain inner setting, divided by expectations before and experiences after implementation.**

**Expectations before implementation**

- Structural characteristics
  - Concerns that there would be no time to complete the additional tasks required to manage the system
  - Concerns that the lack of home care visits would make the system less useful and rarely used

**Experiences after use**

- Structural characteristics
  - With more home care visits, the need for the system increased
- Networks and communications
  - Unclear responsibility and support functions
- Implementation climate
  - Same job, new technology

#### Preimplementation Expectations

##### Overview

There were no mentions of the inner setting during the preimplementation workshops, and predominantly concerns were raised.

##### Structural Characteristics

Staff from the PCCs expressed concerns that the app might not be very useful and that there would be few opportunities for usage due to the few home care visits in primary care. There were also concerns that the management of the app would create additional tasks that there would not be any time for:

*We might not have time for the increased administration.*

[Nurse, MHCO]

### Intervention Experiences

The concerns were mostly addressed, but issues related to the support within the organization were also experienced.

#### Structural Characteristics

Staff who had used the mobile tool for some time reflected that it would be more useful with additional home care visits:

*If I was to have homecare visits a whole day, day after day, then I think it would be good to be able to do all the documentation immediately. Then I think it really would improve patient safety.*

[Nurse, PCC1]

#### Networks and Communications

The responsibility for technical support of the mobile tool and mHealth app was unclear to many of the participating HCP:

*But I don’t know where to turn for help when it doesn’t work. Therefore, I have unfortunately not used it for more than a month.*

[Nurse, PCC2]

Another person stated:

*Well, it’s like; who can you ask?!*

[Nurse, MHCO]

#### Implementation Climate

In many ways, the implementation of the mobile tool did not change the HCP’s perceptions of their work:

*It’s simply a new technology for the same job.*

[Physician, PCC 1]

### Characteristics of Individuals

As previously described, the user’s tolerance for challenges differed. Some continued using the mobile tool despite difficulties, whereas others stopped using it. The categories related to this domain are presented in Textbox 4.

Textbox 4. **Identified categories related to the Consolidated Framework for Implementation Research domain characteristics of individuals, divided by expectations before and experiences after implementation.**

**Expectations before implementation**

- Knowledge and beliefs about the intervention
  - Concerns that the technology will steal time and focus from the patient
  - Concerns of becoming too dependent on the technology
  - Concerns that the project will amount to nothing
- Self-efficacy
  - To dare to try the new technology
- Individual stage of change
  - Looking forward to getting to test
- Individual identification with organization
  - Worry about personal responsibility for the devices

**Experiences after use**

- Knowledge and beliefs about the intervention
  - Positive that this type of intervention could provide benefit
  - Unreliable technology has required patience and persistence
  - Needs further development and additional functions
- Self-efficacy
  - Embarrassing when technology does not work
  - Improved work processes improve patient safety, credibility, and work satisfaction
  - Confidence in using mobile technology
- Individual stage of change
  - Not used to this way of working
  - Creates a need for new knowledge
  - Have not used the technology

#### Preimplementation Expectations

Before the implementation began, users expressed eagerness to test the tools they had been waiting for, despite the already described concerns relating to, for example, internet connectivity and mHealth functionality.

##### Knowledge and Beliefs About the Intervention

HCPs expressed concerns about whether the mobile tool would influence their relationship with the patients, worrying that the technology would shift time and focus from the patient to the technology. They expressed that “there might be too much focus on technology during the visits” (physician, PCC1), and that there could be a “risk for less contact with the patient if the tablet takes more time” (nurse, PCC4).

Another concern expressed was the risk of becoming too dependent on the technology:

*To become dependent on the tablet, and then when it breaks down you don’t know what to do.*

[Nurse, MHCO]

Participants also expressed concerns that the technology might never be ready for use or that its functionality would be reduced to the point that the system would become unusable.

##### Self-Efficacy

Before implementation, the HCPs’ expectations varied. Some were confident in their ability to use it, while others were a bit more hesitant and stressed that daring to try would be important:

*One way is to just start using the tablet; perhaps it’ll go better than we think…*

[Nurse, MHCO]

##### Individual Stage of Change

Similarly, individuals’ readiness to try the technology varied, but most were eager to start testing the mobile tools.

*To dare to start using it and see what happens. I think there are several areas of use. You can find all sorts of online services [mentions Swedish e-health services such as the national patient portal, portals for ordering materials, and online prescription tools]. And perhaps you can help the patient to find the patient portal [1177.se] by using the tablet.*

[Nurse, PCC 3]

Those involved from the start were more positive and eager to test the mobile tools, having waited a long time for the pilot to start, whereas the others were more hesitant.

##### Individual Identification With the Organization

Concerns regarding personal responsibility for the device were only raised during the preimplementation focus groups. Participants had concerns that they might forget the tablet at the patient’s home, drop it, and break the screen. They worried this could be either a waste of resources for the organization or that they would personally be responsible for the costs.

### Intervention Experiences

Although the participants’ characteristics did not necessarily change during the implementation, their self-efficacy and beliefs about the intervention appear to have been affected.

#### Knowledge and Beliefs About the Intervention

The study participants remained positive that the intervention could provide benefits, for example, by making work processes more time efficient and improving patient safety. One of the participants who had used the tool the most said:

*I don’t have to prepare, which usually takes 5 minutes, to print eg, the list of medications, and the latest lab results*

[Physician, PCC1]

Care planning in the patients’ home was also experienced as more efficient:

*I can finish it at the patient’s home, with the patient. You can discuss what to do next, and then that’s done.*

[Physician, PCC 1]

To complete the work while in the field was experienced as reducing the risk of forgetting tasks or losing information:

*[...] and for the patients and the staff [at the nursing home], that they don’t have to remind me, ‘did you remember that? now this hasn‘t arrived, why not?,’ ‘Oh, I didn’t order that, I forgot, or I didn‘t see the note….’ It feels more organized.*

[Nurse, PCC 1]

However, dealing with unreliable technology required patience and persistence from HCP.

*The biggest challenge has been not to lose your patience. It’s like well, now it doesn’t work. I must try the next time again, and the time after that. And that has been difficult since I usually only do this kind of work once a week, so it has been a challenge to not just give up but to instead try and try again.*

[Nurse, PCC 1]

Only the most enthusiastic users had the patience to continue trying despite the unstable technology. The other users, especially those who worked in the MHCO, gave up on the first hurdle and never used the mobile tool.

#### Self-Efficacy

Unstable technology also affects the HCP’s sense of professionalism and could be a cause for embarrassment in front of patients:

*It feels embarrassing when it doesn’t work when you use the tablet. I can live with that, but it’s a little embarrassing. Then it would’ve been better not to have it [the mobile tool]*

[Physician, PCC 1]

One of the HCPs had problems using the mobile tool in the presence of the patient’s relatives:

*That makes me stressed because the technology is new to me. When five people ask me questions at once. I haven’t been able to handle that. [regarding not finding the necessary information on the mobile tool].*

[Physician, PCC 1]

Those who did use the mobile tool found that it improved work processes, patient safety, credibility, and work satisfaction.

*What’s been very positive is that I can finish my work in the patient’s home. I know that I won’t forget anything. The patient won’t be left without medications, which is good. Because it has been stressful before, I haven’t always had the time to finish the work immediately when I’d returned to the workplace… so, I find it to be satisfactory as well, to be able to finish the job and not have any loose threads*.

[Nurse, PCC 1]

They also felt confident using the mobile technology:

*It’s been good. It’s easy to connect and I think it works well. I’ve not experienced any challenges*

[Nurse, PCC 2]

However, there was a significant discrepancy between the two contexts, where the HCPs in the PCCs were much more positive. In the MHCO, most of the HCPs did not use the mobile tool at all.

#### Individual Stage of Change

As mentioned earlier, not all HCPs used the mobile tool:

*I may have a maximum of five [patients]. And then I might as well remember that information or make some notes until I dictate [the record entries]*

[Physician, PCC 1]

One reason was that the users were not used to this way of working. In some cases, the users did not like how record entries were presented:

*I could edit the record entries later if I only write a few keywords [while in the patient’s home]. But I don’t know, I don’t find the entries aesthetically appealing. I think they look much better when I do it my way.*

[Nurse, PCC 2]

In other cases, they had no prior experience writing directly in the medical record, being used to dictating all record entries:

*Well, I don’t have that much experience. I know how to use the iPad, but it feels like I only use it to read record entries, not to write them myself. I usually don’t [write record entries] so I don’t do it here either.*

[Physician, PCC 1]

The users believed there were other possible areas where the mobile tool could be useful:

*I think it’s more that you don’t have enough imagination. You need to find other examples of what you can use it for. You just must use it more and discover even better ways of using it.*

[Nurse, PCC1]

It was believed that it would be better to use the mobile tool regularly to reach its full potential:

*If I had to use this every day, then maybe I would become more comfortable with it*

[Nurse, PCC 1]

### Process

The implementation process was not addressed in interviews with HCP, as this was not the focus of the original project. We will reflect on the implementation process in the discussion.

## Discussion

### Principal Findings

The implementation of the mHealth app was unsuccessful, despite reports of positive experiences and potential benefits. The main reason for the failed implementation was that the app did not sufficiently support the users’ needs. Immature technology and unstable technical infrastructure also contributed. The findings from this implementation study highlight the critical importance of aligning digital health interventions with user needs and existing infrastructure, particularly in the context of aging populations and resource-constrained health care systems. For digitalization to be effective and sustainable, strategic approaches must prioritize usability, cross-organizational compatibility, and user involvement throughout development and implementation. Moreover, adequate resourcing—including time, training, and leadership engagement—is essential to prevent change fatigue and to ensure long-term adoption and impact of mHealth technologies.

Before implementation, HCPs had high expectations that the app would facilitate work processes and reduce cognitive workload by allowing real-time access to information. There were also concerns, mainly due to prior experiences of poor internet connectivity and immature and unstable technology, but also that the app might increase the workload, shift time, and focus from the patient, and that the project would amount to nothing. Ultimately, both expectations and concerns were confirmed. Another preimplementation concern expressed by some HCPs was that they would become too dependent on the new technology. This was not brought up again postimplementation, and we interpret this as being due to the failed implementation. The concern could have negatively influenced the HCP’s willingness to adopt the technology, but likely this was not the most important factor for the failed implementation.

Some users, particularly those involved in the mHealth app’s development, found value in the app despite its shortcomings and kept trying time after time, undeterred by the immature technology. However, users who worked in the MHCO and had not been involved in the design process gave up at the first hurdle and did not use the mobile tool. Data on usage of the mHealth app, for example, the number of log-ins per day or number of active users, were not available during the implementation process. Access to such data could potentially have guided the implementation process so that action could have been taken when usage was low [26].

End user involvement in the design process, often referred to as participatory design [27-29], has proven to be essential in both ensuring that the software meets the end user’s needs and supports their work processes, and increases acceptance among the users [30,31]. In addition to the difference in participation during the development process, the MHCO also used another EHR system in their daily work that was not integrated with the mobile system. The benefit of being able to at least begin documentation in the patient’s home was lost to them, further lowering their incentives to engage with the system. Lack of interoperability and integration between eHealth systems has previously been identified as an important barrier to successful implementation [16]. Physicians who made few home visits and used dictation for EHR documentation in everyday work found the mHealth app less useful. Again, the importance of capturing the perspectives of all potential end users in the design process was highlighted [31,32].

There are several lessons to be learned from this. First, that outcome depends on context, and second, that user engagement in both the development and the implementation processes is crucial for a successful implementation. In our study, both lack of user involvement [30,33] and poor interoperability [7,17] hurt the acceptability of the system, contributing to a system that did not support the user’s work processes [24]. The importance of an interoperable digital health ecosystem is stressed in the World Health Organization digital health strategy, defining it as a “digital interoperable information technology infrastructure that is primarily used by the health care community across all care settings, in particular by health care providers, health service providers and patients” [4]. With such an infrastructure in place, the integration and implementation of the mHealth app would likely have been more successful. The lengthy development and implementation process also affected user engagement negatively. Studies of user involvement’s relationship with user satisfaction have indicated that user involvement can overcome frustration from delays and prolonged projects [30]. In our study, we did see higher user satisfaction and tolerance among users who had been more actively involved in the design process, but it was not enough to overcome the implementation challenges completely. Additionally, for less engaged staff, the barrier to start using a new technology was high, and they also had a greater need for additional support, whereas users with high engagement, often referred to as “champions” in the implementation science literature, kept trying regardless and often found new areas of use for the technology. The most common reason for discarding an eHealth service is issues with the technology [19,34]. eHealth services often are premature and thus do not adequately support the work in home care [19]. Technical issues [19] include everything from a lack of suitable infrastructure to usability issues, the latter referring to whether the product provides the right utility and the degree to which it helps users reach their goals in an effective, efficient, and safe manner, while still being easy to learn and remember how to use [35]. Usability of the mHealth app should have been evaluated more extensively before the implementation, for example, through user-driven scenario-based usability testing [36], or expert- or user-driven heuristic evaluations [37,38].

In our case, considerable problems with internet connectivity, immature technology, and a lack of functionality contributed to the low app usage.

Other studies and reviews confirm the challenges described by the users in this study. User involvement, interoperability, reliability of connection, and technology and infrastructure can be considered facilitators if handled appropriately in the implementation of eHealth, or barriers if they are not considered [12,17]. In a review of 43 studies of mHealth implementation experiences from primary health care services, the main results were that health workers appreciated mHealth when it improved feedback, speed, and workflow [17]. Challenges stated were that mHealth sometimes created more work, resulting in some preferring paper instead. Some health workers found the decision support opportunities useful, and others thought it threatened their clinical skills [18]. The aspect of workflow was found substantial for the success or failure of eHealth interventions particularly if workload increased, if workflow was interrupted, and if there was an alignment with clinical processes [17].

### CFIR Suitability for mHealth Implementation

CFIR is developed to study implementation processes, not specifically new technology. We chose to include “technical infrastructure” as a new construct in the domain “Outer setting” since the effect of new technology depends on its functionality. A more specific focus on the usability of the eHealth or mHealth intervention could also be included under intervention characteristics, but we chose to use the “design quality” construct here.

The implementation process is crucial for the success of an implementation and for a successful implementation, all stages of the implementation must be properly executed. Our study was not originally designed to study the implementation process, and subsequently, material on some aspects of the implementation process is missing since no questions regarding those aspects were asked in the interviews. We see, however, that the implementation of the mobile tool most likely would have benefited from better planning, execution, support functions, and resources. When implementing complex interventions, feasibility and pilot studies are essential [35,39]. This is especially important when implementing mHealth where lack of usability is often the cause of failed implementations. This could be further specified in the CFIR domain “process” when studying new technology. A specific challenge of implementing the mHealth app in a rural home care area was the physical distance between units and the challenges of keeping day-to-day contact with and providing support to the HCP. The need for a specific facilitator role, that is, a person dedicated to supporting the implementation process and ensuring problems are identified and addressed, was apparent, as is also stressed in other implementation theories, for example, the Promoting Action on Research Implementation in Health Services (PARIHS) framework [40]. In line with the integrated-Promoting Action on Research Implementation in Health Services (i-PARIHS) framework and other implementation models, the facilitator role typically requires a combination of technical, organizational, and interpersonal competencies. Core competencies include basic technical troubleshooting and familiarity with the intervention, skills in user training and coaching, and the ability to communicate effectively across different organizational levels, including leadership. Responsibilities often span the entire implementation process: supporting planning and readiness activities before rollout, providing hands-on assistance and problem-solving during implementation, and ensuring ongoing support and feedback loops during maintenance. While the exact scope and emphasis of these tasks must be adapted to the local context and resources, these elements are commonly highlighted as critical for successful implementation.

There is a strong belief in the potential for eHealth and the introduction of new technology to revolutionize health care and that it will be necessary for health care and primary care in the future [4]. The introduction of new technology, however, often fails [17,41], and there is a risk that premature implementation of new technology will exhaust HCP for future projects and implementation. The challenges and concerns must be considered to move forward successfully [15]. According to the CFIR [23,42] and i-PARIHS [40] frameworks, the individuals’ previous experiences and beliefs about the intervention will impact the implementation’s success. When HCPs are exposed to immature digital solutions with poor usability, we may risk a negative impact on future implementation and innovation projects. To avoid this, we conclude that it is important to ensure that new technology has the necessary functionality and stability before implementation and that there are routines in place in the organization to guide decision-making and implementation processes. Structured contingency planning for technical failures, such as providing clear fallback procedures, timely support, and communication strategies, could sustain user engagement by reducing frustration, maintaining workflow continuity, and reinforcing trust in the implementation process despite technological setbacks.

### Strengths and Limitations

The study was guided by the CFIR framework, which is a well-established model for understanding implementation processes [23]. This adds theoretical rigor and structure to the analysis, ensuring that a broad range of influential factors is considered. In our analysis, we used the original CFIR framework which was available when we began our analysis. More recent versions of the framework [42] have added new constructs; however, the domains remain the same, and we believe that using the most recent version of CFIR would not have substantially influenced our results.

The use of interviews (both individual and focus group) allowed for an in-depth exploration of the HCPs’ experiences and perspectives. This is particularly useful given the small number of users of the mHealth app, making qualitative methods appropriate for capturing rich, detailed insights. The study also collected data from both nurses and physicians across different settings (PCCs and MHCO), which ensured a variety of perspectives were included in the analysis. The small sample size (8 nurses and 1 physician from each of the two groups) may limit the generalizability of the findings to other settings or health care systems. Additionally, the majority of participants were female, which may impact the diversity of perspectives, yet it reflects the current workforce in Swedish home care. To maintain anonymity, the study does not report detailed demographic information about participants, such as years of experience, which could be useful for understanding how these factors might influence perceptions of the mHealth app. We also do not include any quantification of responses, which means that while rich qualitative data were captured, the relative importance of different factors or themes was not assessed. This could limit the ability to weigh certain barriers or facilitators more heavily than others; however, considering the qualitative nature of our dataset, we argue that this is the best approach as quantifying the results may be rather misleading. Using a validated instrument to measure health care professionals’ self-efficacy could be a way to further understand how self-efficacy impacts adoption and abandonment, and how it may change over time during an implementation project. This should be explored further in future research.

A strength of the study is that the data collection spanned several years, and follow-up interviews after 6 months of using the mobile tool captured longer-term experiences and effects, offering insights into sustained usage and challenges. However, the study took place from 2015 to 2017, with final data collection in 2018, which could render the results of the study out of date. Yet the findings remain highly relevant in today’s digital health landscape. Despite technological advancements, the fundamental implementation challenges we identified, such as poor alignment with clinical workflows, limited user engagement, inadequate training and support, and insufficient organizational readiness, persist in contemporary mHealth and eHealth initiatives. Implementation projects continue to experience implementation challenges across diverse settings, also in large-scale initiatives. For example, recent EHR rollouts in Sweden [43], Norway [44], and Finland and Denmark [45] encountered major disruptions due to user dissatisfaction, lack of preparation, and technological shortcomings. These cases underscore the enduring importance of addressing the sociotechnical and organizational dimensions of digital health implementations.

The first author was involved in the needs and requirement analysis for the mHealth app and also conducted the interviews. This dual role might introduce potential bias into the data collection and analysis process, as prior involvement may influence how the data are interpreted or what aspects are emphasized. Throughout the analysis process, we have been vigilant about this, and the involvement of Master’s students with no prior connection to the project facilitated an unbiased data collection. The first author’s familiarity with the context and the app has also been an enabling factor, to both data collection and by facilitating insights into both the design and implementation processes that would otherwise have been challenging to obtain in retrospect.

These strengths and limitations provide important context for interpreting the findings of the study and considering its applicability to broader implementation efforts.

### Conclusions

We conclude that new technology must be stable and have the desired functionality before implementation for success. The functionality of a new technology must support the users’ needs and be sufficiently integrated with other IT systems in the health care organization. How the implementation process is executed is important, but the implementation will not be successful if the new technology does not properly support the users’ needs. In the case presented here, implementation failed because the technology was unstable and thus unreliable, not sufficiently supportive of the users’ needs, and not adapted to the users’ workflow. There were, however, positive experiences too, and mHealth still has a strong potential to support HCPs working in home care. To achieve this potential, we must ensure that sufficient resources are allocated to both design and development, evaluation and feasibility studies, and support and engagement during the implementation of mHealth in the future.

## Supplementary material

10.2196/69590 Multimedia Appendix 1 Screenshots of the mobile health app.

10.2196/69590 Multimedia Appendix 2 Interview guide focus group interview—expectations of the intervention.

10.2196/69590 Multimedia Appendix 3 Interview guide—evaluation after the intervention.

## Abbreviations

CFIR: Consolidated Framework for Implementation Research
EHR: electronic health record
HCP: health care professionals
i-PARIHS: integrated-Promoting Action on Research Implementation in Health Services
MHCO: municipality home care organization
mHealth: mobile health
PARIHS: Promoting Action on Research Implementation in Health Services
PCC: primary care center
