# Supplementary material for: Failed Implementation of Mobile Access to Electronic Health Records in Home Care: Qualitative Study in Sweden
Source: JMIR Mhealth Uhealth. 2026 Jan 23;14:e69590. doi: 10.2196/69590 (PMC12829896; doi:10.2196/69590)

**Appendix 4** – Example screenshots from the mHealth app (in Swedish)

The screenshots only contain test data.


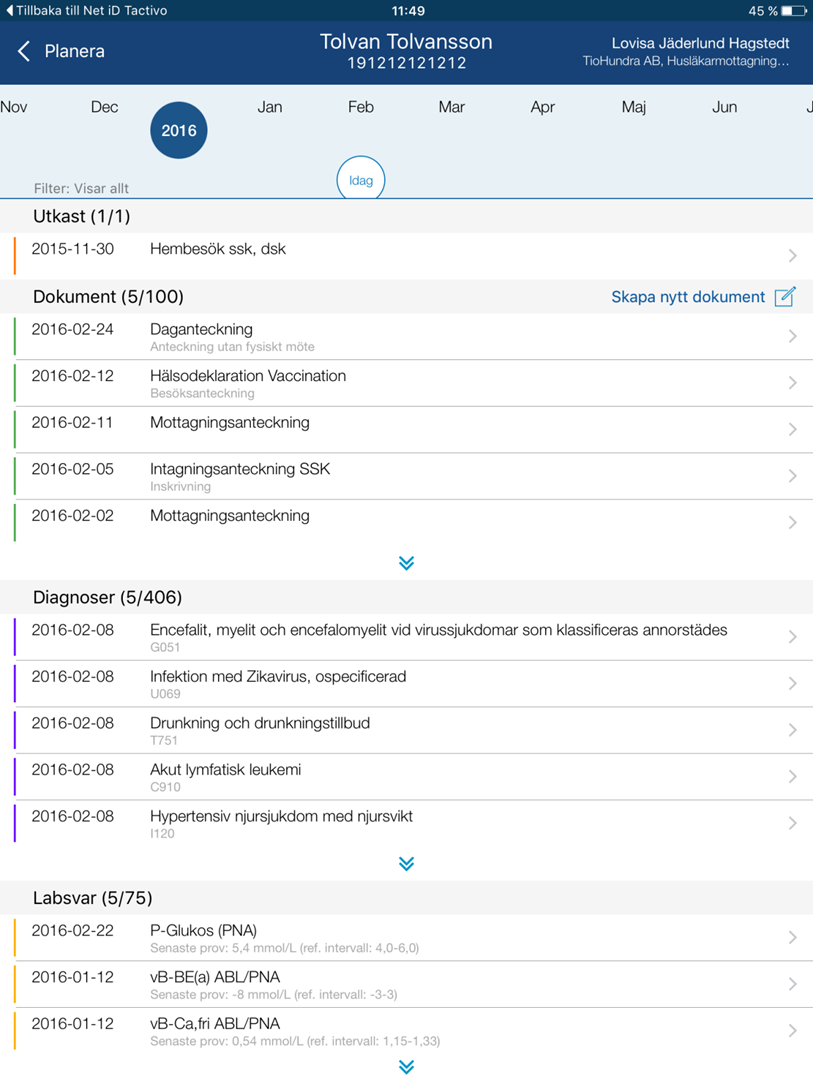


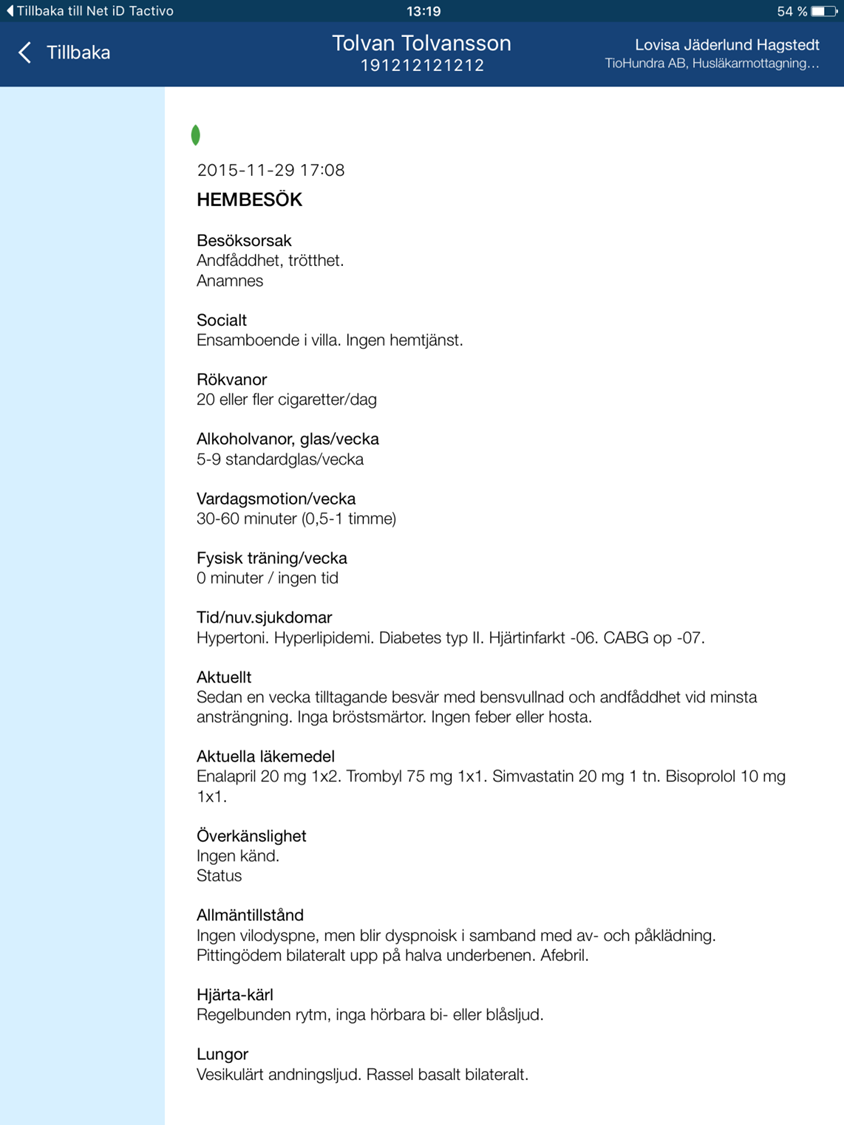

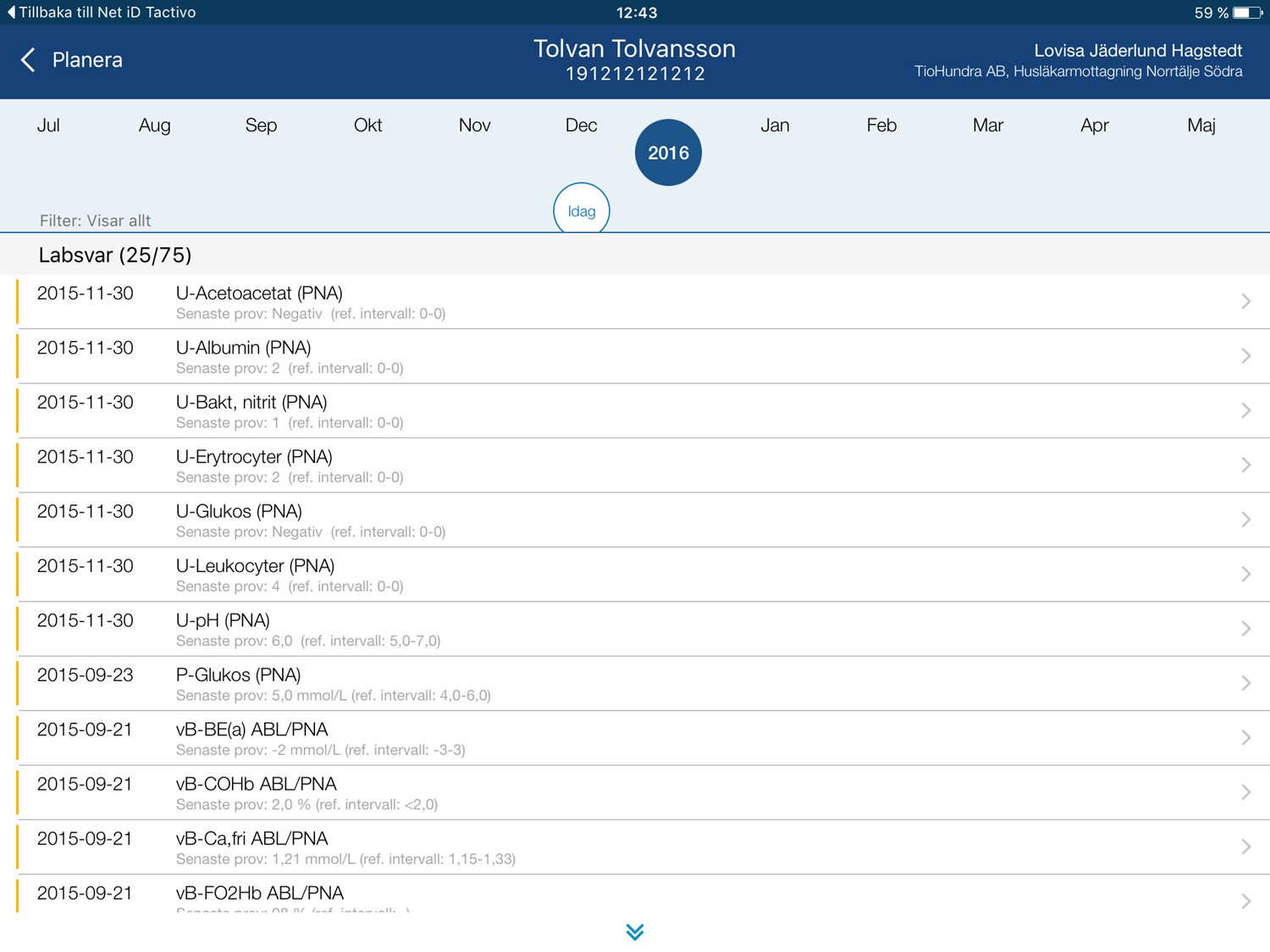

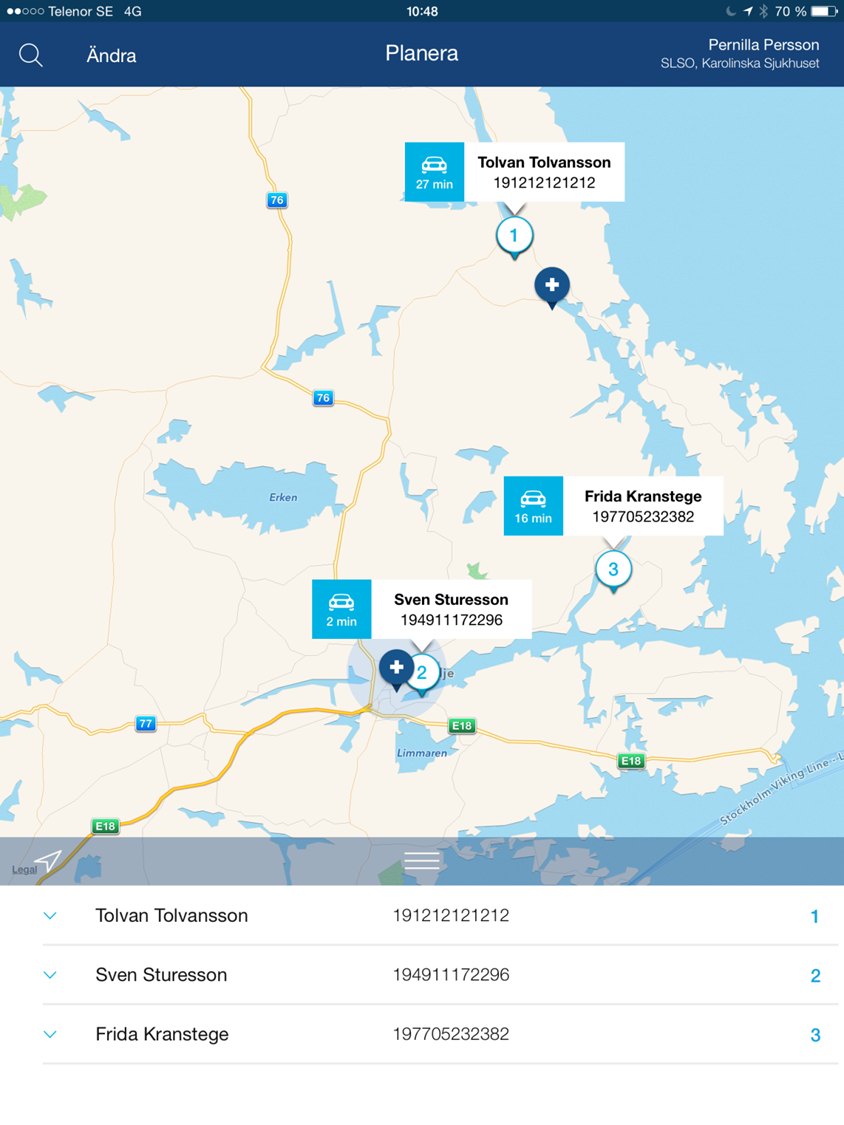

Supplement: Multimedia Appendix 1 [file mhealth-v14-e69590-s001.docx]
