# Supplementary material for: Failed Implementation of Mobile Access to Electronic Health Records in Home Care: Qualitative Study in Sweden
Source: JMIR Mhealth Uhealth. 2026 Jan 23;14:e69590. doi: 10.2196/69590 (PMC12829896; doi:10.2196/69590)
Supplement: Multimedia Appendix 2 [file mhealth-v14-e69590-s002.docx]

**Appendix 1**

**Interview guide focus group interview – expectations of the intervention**

1. What are your expectations of starting to use the mobile tool?

Positive? Negative?

Give examples ...

(first document individually on post-it notes - then discuss in the group)

(implementation process, management communication, training, technical support, etc.).

2. What problems in your daily work do you hope to solve by using the mobile tool?

(quality of documentation, access to information, navigation support, communication with other care providers, communication with patients, clinical decision support systems)

3. How would you like to solve / counteract the fears that have been raised in the group?

(start from the compiled post-it notes and try to come up with an action plan to facilitate the introduction of the mobile tool)
