# Supplementary material for: Failed Implementation of Mobile Access to Electronic Health Records in Home Care: Qualitative Study in Sweden
Source: JMIR Mhealth Uhealth. 2026 Jan 23;14:e69590. doi: 10.2196/69590 (PMC12829896; doi:10.2196/69590)
Supplement: Multimedia Appendix 3 [file mhealth-v14-e69590-s003.docx]

**Appendix 2**

**Interview guide - evaluation after the intervention**

1. What has it been like to start using the mobile tool in your daily work?

What has been positive? What has been negative?

Give examples…

(Concerning implementation process, management communication, training, technical support, etc.).

What difficulties / challenges have you experienced in starting to use the mobile tool?

What benefit / benefits have you experienced from starting to use the mobile tool?

2. How have access to information about patients in the mobile tool affected your work?

Is there any information you get access to that you feel you do not use / need?

Is there any information you feel you have not had access to?

3. How have access to the map function in the mobile tool affected your work?

Have you benefited from being able to navigate more easily to patients? To be able to see the location of colleagues?

4. How has the possibility to use the mobile tool for documentation in the electronic health record, while on home visits, affected your work?

What has been positive? What has been negative?

Give examples…

How do you feel about documenting?

When you are in the patient’s home?

When you are between home visits?

When you're in the office?

Quality of documentation, patient safety…

5. How have your communication with others been affected? Within the working group, towards other care providers, towards patients, towards patients’ relatives.

What has been positive? What has been negative?

Give examples…

6. Do you use the mobile tool for something other than the mHealth application?

Video communication, clinical decision support/prescription systems,

email, photos

7. Would you recommend other colleagues to use the same or a similar mobile tool? Why?

8. Is there anything you would like to change / improve with the mobile tool?
